# ZO-2 Is a Master Regulator of Gene Expression, Cell Proliferation, Cytoarchitecture, and Cell Size

**DOI:** 10.3390/ijms20174128

**Published:** 2019-08-24

**Authors:** Lorenza González-Mariscal, Helios Gallego-Gutiérrez, Laura González-González, Christian Hernández-Guzmán

**Affiliations:** Center for Research and Advanced Studies (Cinvestav), Department of Physiology, Biophysics and Neuroscience, Mexico City 07360, Mexico

**Keywords:** tight junctions, ZO-2, cholestasis, gene transcription, hypertrophy, tumor suppressor, NLS, NES, CaSR, RhoA

## Abstract

ZO-2 is a cytoplasmic protein of tight junctions (TJs). Here, we describe ZO-2 involvement in the formation of the apical junctional complex during early development and in TJ biogenesis in epithelial cultured cells. ZO-2 acts as a scaffold for the polymerization of claudins at TJs and plays a unique role in the blood–testis barrier, as well as at TJs of the human liver and the inner ear. ZO-2 movement between the cytoplasm and nucleus is regulated by nuclear localization and exportation signals and post-translation modifications, while ZO-2 arrival at the cell border is triggered by activation of calcium sensing receptors and corresponding downstream signaling. Depending on its location, ZO-2 associates with junctional proteins and the actomyosin cytoskeleton or a variety of nuclear proteins, playing a role as a transcriptional repressor that leads to inhibition of cell proliferation and transformation. ZO-2 regulates cell architecture through modulation of Rho proteins and its absence induces hypertrophy due to inactivation of the Hippo pathway and activation of mTOR and S6K. The interaction of ZO-2 with viral oncoproteins and kinases and its silencing in diverse carcinomas reinforce the view of ZO-2 as a tumor regulator protein.

## 1. Introduction

Zonula occludens proteins ZO-1, ZO-2, and ZO-3 belong to the membrane associated guanylate kinase homologue (MAGUK) protein family and are concentrated at the cytoplasmic face of tight junctions (TJs) in epithelial cells. Although these proteins share a core of common structural and functional domains and interacting partners, they also exhibit particular features that give rise to certain non-redundant functions among these proteins (for review see [[1,2]]). This review concentrates on ZO-2 and its non-canonical role in gene transcription, cell proliferation, and modulation of cell size, cytoarchitecture, and cancer.

ZO-2 is a 160 kDa protein, first identified by its co-immunoprecipitation with ZO-1 in epithelial cells [3]. ZO-2 is encoded by the *TJP2* gene located on human chromosome 9 q21.11 [4]. ZO-2 is present at TJs but in non-epithelial cells like fibroblasts that lack TJs, ZO-2 concentrates at adherens junctions (AJs) [5]. In cardiac muscle cells, the observations are contradictory. Some report the presence of ZO-2 in co-localization with ZO-1 at specialized intercellular junctions, known as fascia adherens or intercalated discs, which connect the opposing ends of cardiac muscle cells [5]. Others indicate that only ZO-1 is present at fascia adherens [6] and that ZO-2 has a diffuse cytoplasmic distribution in myocardium tissue [7].

**Figure 1:**
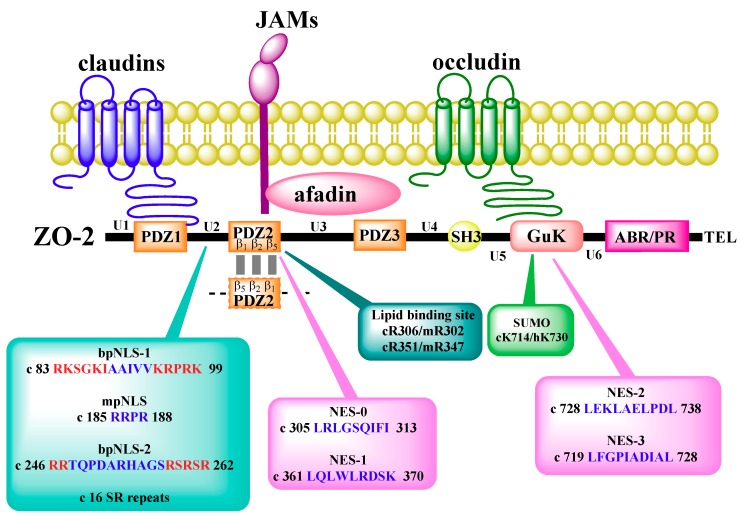
ZO-2 molecular organization and interactions with integral tight junction (TJ) proteins at the plasma membrane. ZO-2 domains (PDZ, SH3, and GuK), regions (U, unique; ABR, actin binding; PR, proline rich), and PDZ-binding motif (TEL) are indicated, as well as the nuclear localization signals (NLS) and exportation signals (NES), SUMOylation (SUMO) and lipid binding sites, and dimerization region. The ZO-2 sequence is identified by letters: c, canine; m, mouse; h, human. Numbers correspond to amino acids. Clusters of basic amino acids (K/R) in the bpNLS are shown in red. bp, bipartite; mp, monopartite.

ZO-2 is a scaffold protein, whose amino segment, containing PDZ1-3-SH3-GuK domains, binds to integral and peripheral proteins of the TJs, including occludin, claudins, JAM-A, cingulin and ZO-1, to proteins of the AJs, like α-catenin and β-catenin, and to gap junction connexins (for review see [8]). Instead, the carboxyl segment of ZO-2, which exhibits the acidic and proline rich regions and ends with a motif that binds PDZ (PSD95, Dlg1 and ZO-1) domains, distributes when separately introduced into epithelial cells, along actin filaments [5] (Figure 1).

PDZ1-3 modules, SH3 and GuK domains, and the acidic region of ZO-2 display a high percent of identity and similarity to those in other ZO proteins, with ZO-1 having a higher percent of both than with ZO-3 [9]. In situ ZO-2 is present as a ZO-1/ZO-2 complex, but not in ZO-2/ZO-3 or ZO-1/ZO-2/ZO-3 complexes [10]. The high conservation present between PDZ2 domains in ZO proteins allows these PDZ domains to dimerize via three-dimensional domain swapping, generating heterodimers of ZO-1-PDZ2/ZO-2-PDZ2 and ZO-1-PDZ2/ZO-3-PDZ2 domains only, as well as homodimers of ZO-1-PDZ2 and ZO-2-PDZ2 domains only [11]. Structural analysis of ZO-2 PDZ2 revealed that it has five β sheets and two α helices and that ZO-2-PDZ2 homodimers form by the interaction of three antiparallel β sheets, β1-β5’, β1′-β5, and β2-β2′, due to extensive inter-subunit hydrogen bonds and hydrophobic interactions. In addition, chemical crosslinking and dynamic laser light scatter experiments revealed that ZO-1-PDZ2 and ZO-2-PDZ2 form oligomers in solution. This oligomerization mediated by PDZ2 domains in ZO-1/ZO-2 proteins might provide a scaffold for the assembly of TJs.

Both ZO-1 and ZO-2 independently allow the polymerization of claudins and determine the site of TJ strand formation [12]. Thus, epithelial cells lack TJs when ZO-1 and ZO-2 expression is suppressed, and when either of these proteins is exogenously expressed, claudins polymerize and TJ filaments are again observed in freeze-fracture replicas. However, when a truncated segment of ZO-1 was introduced containing only the PDZ1-3 domains, it localized in the cytoplasm, not in the membrane, and the claudins did not polymerize. However, when a longer construct consisting of PDZ1-3 and SH3–GuK domains was introduced, TJ strands formed. The importance of the SH3–GuK segment is highlighted by the fact that it also plays a role in the dimerization of MAGUK proteins and binds to the AJ proteins afadin and α-catenin, allowing the recruitment of ZO-1 to the proximity of the plasma membrane. If in ZO-1 knock out (KO)/ZO-2 knock down (KD) cells the amino PDZ1-3 segment of ZO-1 was forcibly recruited to the membrane by the addition of a myristoylation signal, no claudin polymerization happened. Instead, if this construct was allowed to dimerize by the introduction of a FK506-binding protein (FKBPv) signal and a homodimerizer, claudin polymerization was abundant throughout the lateral membrane, highlighting the crucial importance of ZO-1 dimerization for claudin polymerization. Although the latter experiments were done with ZO-1, the same results were expected to occur with ZO-2, since their MAGUK domains are so similar. Taken together, these observations suggest that claudin binding through the PDZ1 domain of ZO-1 or ZO-2, as well as the SH3–GuK mediated recruitment of these proteins to the AJ and the dimerization of ZO-1 and ZO-2, allow the polymerization of claudins at the TJ region.

## 2. ZO-2 During Early Development

In mouse preimplantation embryos, ZO-2 assembles at blastomere junctional sites at the 16-cell stage, at the same time as cingulin but later than ZO-1α^-^, rab13, and the Par-3/Par-6/aPKC complex, which reach the junctions after the compaction of 8-cell morulae. At the 16-cell stage, ZO-2 co-localizes with E-cadherin in a transient complex that contains proteins of both the TJ and the AJ. From the 32-cell stage onwards, ZO-2 and the rest of the TJ proteins, also including occludin and claudins, separate from those of the AJ and a blastocoel cavity is formed [13] (for review see [14]). Lack of ZO-2 in the trophectoderm of mouse preimplantation embryos delays the formation of the blastocoel cavity and induces an increased assembly of ZO-1, suggesting a compensatory mechanism of ZO proteins. In accordance, the double KD of ZO-1 and ZO-2 induces a more severe inhibition of blastocoel formation [13]. Early after implantation, KO of ZO-2 is lethal and mouse embryos die, showing decreased proliferation at embryonic day 6.5 (E6.5) and increased apoptosis at E7.5 [15]. However, embryonic lethality of ZO-2 KO mice was rescued by injecting ZO-2 KO embryonic stem cells into wild type blastocysts to generate viable ZO-2 chimeras, hence indicating that ZO-2 is crucial for extraembryonic tissue rather than for the development of the embryo *per se* [16].

## 3. ZO-2 Presence at TJs Is Crucial in the Mouse Testis and the Human Liver and Inner Ear

ZO-2 is present in the TJs of all epithelial cells and has a role on the barrier function of TJs, as ZO-2 KD cells achieve a much lower peak value of transepithelial electrical resistance (TER) than parental cells [17,18]. Therefore, the observation that the absence of ZO-2 causes severe barrier impairment in mouse testes and human liver and inner ear tissue suggests that compensatory mechanisms, like the over-expression of ZO-1, are not effective in these tissues, where ZO-2 plays a critical and non-redundant role (Table 1).

### 3.1. ZO-2 and the Blood–Testis Barrier

TJs form the blood–testis barrier (BTB) at the lowermost portion of the lateral membrane of Sertoli cells. This barrier divides the paracellular space in two compartments: The basal compartment, which contains diploid spermatogonia, and the adluminal compartment, which contains differentiating spermatocytes and haploid spermatids. The BTB, constituted by occludin, claudin-3 and -11, ZO-1, and ZO-2, is critical for the maintenance of a proper microenvironment for germinal cells and to prevent autoimmunity (for review see [29]). Male ZO-2 chimera mice generated from wild type blastocysts, in which ZO-2 KO embryonic stem cells substituted the normal stem cells, showed reduced fertility, smaller testes, degeneration of seminiferous tubules, and permeable BTB, even when the other TJ proteins were present at the BTB. This suggests that ZO-2 plays a unique and critical role in the BTB [16].

Moreover, the organophosphate pesticide methamidophos (MET), which is widely used in agriculture in developing countries and is known to cause adverse effects in human male reproductive function, opens the BTB of mice and perturbs spermatogenesis. These changes correlate with MET formation of covalent bonds with ZO-2 in serine, tyrosine, and lysine residues. MET bonds formed in ZO-2 are proposed to interfere with ZO-2 degradation and TJ sealing, since cultured Madin-Darby canine kidney (MDCK) cells transfected with ZO-2 mutated at MET target sites developed lower values of TER [30].

### 3.2. ZO-2 in the Liver

In the liver, TJs present in hepatocytes and cholangiocytes in intrahepatic canaliculi prevent leakage of biliary components through the paracellular space and into the liver parenchyma. Familial hypercholanemia is a rare disease characterized by elevated serum bile acid concentration, pruritus, and fat malabsorption. In Amish individuals with this condition, the missense mutation V48A in the first PDZ domain of TJP2 was identified. This mutation reduces PDZ1 domain stability and binding to the C-terminal domain peptides of claudin-1, -2, -3, -5, and -7. In some individuals, this mutation is associated with an additional missense mutation on the gene for bile acid Coenzyme A (BAAT), a bile-conjugating enzyme. In a patient homozygous for the *TJP2* mutation and heterozygous with respect to the *BAAT* mutation, TJ depth viewed by transmission electron microscopy was greater than in control individuals. Considering that greater TJ depth was observed in liver disease associated with greater TJ permeability, it was concluded that the missense mutation in *TJP2* generated leakier TJs in the liver, which affected bile acid localization [21].

In children with familial intrahepatic cholestasis, a condition where bile does not flow through the intrahepatic ducts of the liver, homozygous protein truncating mutations in *TJP2* leading to a complete absence of the protein were detected. In this case, the absence of ZO-2 is accompanied by a failure of localization of claudin-1, but not of claudin-2, to the canalicular membrane [31]. Another case of compound heterozygous mutations in *TJP2* predicted to abolish TJ protein translation was observed in a young child with familial intrahepatic cholestasis [24]. Additional cases of infants with progressive intrahepatic cholestasis that led to chronic cholestatic hepatitis with cirrhosis and hepatocellular carcinoma were also reported. In these cases, homozygous and heterozygous *TJP2* mutations were present, ZO-2 expression was absent, and claudin-1 expression was decreased [25,26,27].

Intrahepatic cholestasis of pregnancy, characterized by pruritus, hepatic impairment, and elevated serum bile acids, can lead to spontaneous preterm delivery and stillbirth. In women with this condition, heterozygous mutations in *TJP2* were discovered. These novel T62M and T626S mutations were respectively located in the PDZ1 and the SH3 domains of ZO-2; and no patient harbored more than one mutation [28]. Since only three patients were identified with these mutations, larger cohorts of pregnant patients should be studied to understand why these variants give rise to leaky TJs in the liver during pregnancy (Table 1).

### 3.3. ZO-2 in the Inner Ear

In the mammalian ear, the hair cells of the cochlea convert the vibrations induced by sound into electrical signals by K^+^-mediated depolarization. A K^+^-rich fluid, named endolymph and produced by the stria vascularis, bathes the apical surface of hair cells. The basolateral surface of a hair cell is surrounded by the perilymph, which has a low K^+^ concentration. To prevent mixing endolymph and perilymph and to maintain a high resting potential in the endolymph (≈90 mV), the cells surrounding the liquid are sealed with TJs, which are very tight in hair cells and supporting cells in the reticular lamina of the organ of Corti and in some cells of the stria vascularis. Accordingly, hearing loss associated with the degeneration of cochlear hair cells was observed in KO mice for claudin-14 [32], occludin [33], tricellulin [34], and ILDR-1/angulin-2 [35], and in a mice carrying a missense mutation of claudin-9 [36]. Claudin-11-null mice lacked TJs in the basal cells of the stria vascularis and their deafness was associated with a low endocochlear potential [37,38].

Autosomal dominant nonsyndromic hearing loss caused by mutations in the *TJP2* gene was observed in a Chinese family involving the missense mutation 2081G>A (G694E) [20], located within the GuK domain of ZO-2, and in the Korean population due to two pathogenic variations: 334G>A (A112T), which is present in the PDZ1 domain of ZO-2, and 3562A>G (T1188A), which is situated within the carboxyl terminal PDZ binding motif TEL of ZO-2 [19]. Since the latter, as well as the PDZ1 and GuK domains of ZO-2, are protein–protein binding regions, it is expected that mutations at these sites would compromise TJ stability and the interaction of ZO-2 with other TJ proteins and signaling molecules (Table 1).

## 4. Subcellular Localization and Traffic of ZO-2

ZO-2 can be found in the cytoplasm, nuclei, and TJs. In unfertilized mouse eggs, ZO-2 distributes diffusely in the cytoplasm. In the zygote, ZO-2 concentrates in specks around both pronuclei. After the first cell division these specks appear in the cytoplasm close to the plane of cleavage. From late 2-cell embryos until 8-cell compaction, ZO-2 concentrates at the nuclei of the blastomeres, but is excluded from the nucleoli. After 8-cell compaction, nuclear ZO-2 diminishes in intensity and instead concentrates at junctional sites from the 16-cell stage onward [13].

**Figure 2:**
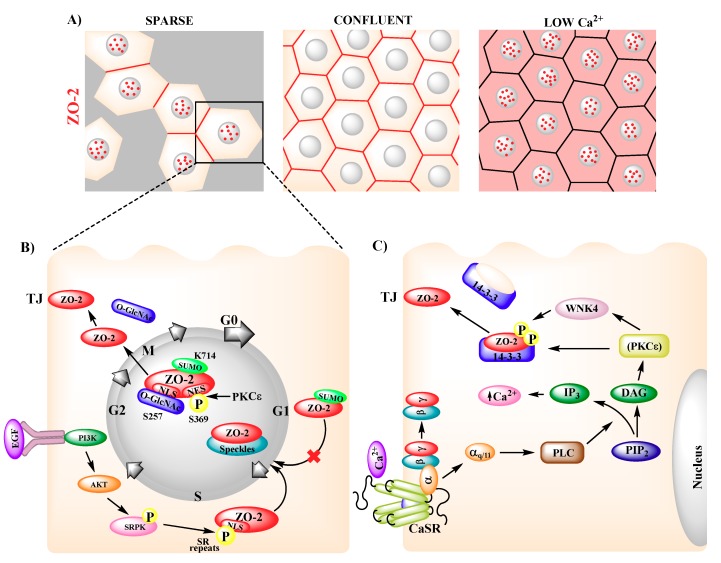
Intracellular movement of ZO-2. (**A**) In sparse cultures, ZO-2 is present in nuclear speckles (red dots) and TJs (red cell borders), whereas in confluent cells ZO-2 concentrates at TJs (red cell borders). In cells cultured with low Ca^2+^ (1–5 μM) ZO-2 distributes diffusely in the cytoplasm (pink cytoplasmic staining) and concentrates in nuclear speckles (red dots). (**B**) In sparse cultures, epidermal growth factor (EGF) induces AKT activation, which triggers ZO-2 phosphorylation of serine-arginine (SR) repeats by SR protein kinase (SRPK). Nuclear localization signals (NLSs) of ZO-2 and phosphorylated SR repeats induce the arrival of ZO-2 to nuclear speckles at late G1. ZO-2 exit from the nucleus requires the phosphorylation of Ser369 within a nuclear exportation signal (NES) by PKCԑ and the inactivation of bpNLS-2 by *O*-GlcNAc of Ser257, and is facilitated by SUMOylation of Lys714. (**C**) Extracellular Ca^2+^ activates the Ca^2+^-sensing receptor (CaSR) present in the plasma membrane that signals through the Gα_q/11_ subunit, activating PLC, which hydrolyzes PIP2 into DAG and IP3. DAG activates PKCԑ, leading to ZO-2 phosphorylation by WNK4. This phosphorylation induces the disassembly of ZO-2 from 14-3-3 proteins in the cytoplasm and triggers the relocation of ZO-2 to the plasma membrane.

In confluent epithelial cells, ZO-2 concentrates at the TJs, whereas in sparse cultures, ZO-2 is present both at the nucleus and TJs [39], thus revealing that the localization of ZO-2 is regulated by the degree of cell–cell contact (Figure 2A). Nuclear recruitment of ZO-2 can also be induced at sites of monolayer wounding [39] or with environmental stresses, such as heat shock (42 °C) and chemical insult (CdCl_2_) [40].

ZO-2 stability augments with TJ establishment and maturity. Thus, the half-life of ZO-2 in epithelial MDCK cells goes from 7 h in cells cultured in media with low Ca^2+^ (1–5 µM) that cannot form TJs, to 19.7 h in cultures with 1.8 mM Ca^2+^ [41] and from 8.6 h in sparse cultures to 19.1 h in confluent cultures [3]. 

In MDCK cells, treatment with L-mimosine, which selectively blocks cell cycle progression in the late G1 phase, revealed that ZO-2 enters the nucleus at the later stages of G1, while treatment with nocodazole, which arrests the cell cycle at the beginning of mitosis, showed that ZO-2 exits the nucleus during mitosis [42]. These observations explain why the nucleus is devoid of ZO-2 in confluent quiescent cells, while proliferating cells have ZO-2 at the nucleus (Figure 2B).

Nuclear importation of ZO-2 relies on nuclear localization signals (NLSs) located within the U2 region of the molecule, between PDZ1 and PDZ2 [43]. The first bipartite (bp) NLS of ZO-2 is conserved in several species, including human, dog, mouse, and chicken, and is functional in a reporter protein nuclear importation assay. The second bpNLS of the molecule, present in dog and mouse, is not active in this assay [44]. The functionality of the second bpNLS is hampered by the phosphorylation of three serine residues (S257, S259, and S261) present within the carboxyl segment of the signal, which neutralizes the positive charges of three contiguous arginine residues (Figure 1).

In addition, the U2 region of ZO-2 has 16, 14, 19, and 16 serine–arginine (SR) motifs in dog, human, mouse, and chicken, respectively. SR motifs are also present in transcription factors and pre-mRNA splicing factors and constitute a signal necessary and sufficient to target proteins for nuclear speckles. ZO-2 also has a docking motif for SR protein kinase 1 (SRPK1) in its U2 domain that specifically phosphorylates serine residues present in SR motifs. Accordingly, ZO-2 associates to SRPK1 and the serine phosphorylation of ZO-2 by SRPK1, which is triggered by the presence of EGF and the subsequent activation of PI3K and AKT, induces the translocation of ZO-2 into the nucleus and its distribution in speckles [44] (Figure 2B). The binding of the PDZ2 domain of ZO-2 to phosphatidylinositol 4,5-bisphosphate (PIP2) regulates ZO-2 recruitment to nuclear speckles, and ZO-2 silencing disrupts speckle morphology, suggesting that ZO-2 plays an active role in the formation and stabilization of nuclear speckles. Residues R302 and R347 in the PDZ2 of mouse ZO-2 are critical for PtdIns binding [45].

ZO-2 exits the nucleus as the monolayer acquires confluence through a process sensitive to leptomycin B, a drug that blocks the interaction between nuclear exportation signals (NES) and exportin1/CRM1 [39]. NES are leucine-rich sequences containing a characteristic spacing of leucine or other hydrophobic residues. Based on the archetypal NES consensus sequence of HIV-1, Rev, PKI-α, IκBα, and zyxin, four NES in ZO-2 canine sequences were identified, three in each of mouse and chicken, and none in human ZO-2. In canine ZO-2, two NES are located at the PDZ-2 domain and the other two at the GuK module (Figure 1). With reporter protein nuclear export assays, the functionality of three of these signals was confirmed, while that of NES-1 proved to be effective only after a phosphomimetic mutation was introduced in a serine residue (Ser369) within the signal that constituted a putative protein kinase C (PKC) phosphorylation site [46] (Figure 2B). Although each of the four NES present in ZO-2 can export a reporter protein like ovalbumin, in full-length ZO-2 the presence of the four intact NES is required to avoid nuclear accumulation of the protein [46]. Since Ser369 is phosphorylated by nPKCε, this phosphorylation constitutes a critical step for the exit of ZO-2 from the nucleus [47].

Two additional post-translational modifications facilitate the nuclear exportation of ZO-2. One is the *O*-linked β-*N*-acetylglucosamination of ZO-2 at Ser257. Since this residue is located within the second bpNLS of ZO-2, it is proposed that this modification inactivates the NLS and consequently facilitates the departure of ZO-2 from the nucleus [44]. The other modification is the SUMOylation at Lys730 of human ZO-2, as mutation of this site to arginine results in prolonged nuclear localization of ZO-2 in nuclear recruitment assays. In addition, a construct mimicking constitutive SUMOylation of ZO-2, preferentially localized in the cytoplasm, suggesting that SUMOylation in ZO-2 occurs in the nucleus and is a signal that inhibits entry to the nucleus and instead facilitates nuclear exportation [48] (Figure 2B).

Newly synthesized ZO-2 first appears at the nucleus of sparse cells and as the monolayer becomes confluent, it relocates from the nucleus to the plasma membrane [39]. Thus, at the earliest times after transfection, around 80% of ZO-2 transfected cells showed ZO-2 at the nucleus; this value diminished to 20% after 18 h. With the help of a nuclear microinjection assay, in which antibodies against ZO-2 were introduced into the nucleus, it was observed that newly synthesized and endogenous ZO-2 made a stopover at the nucleus before reaching the plasma membrane [47]. The nucleus seemed to serve as a cellular reservoir of ZO-2, as the amount of ZO-2 in this compartment was significantly higher than that which eventually reached the plasma membrane. In the latter, the amount of ZO-2 stayed relatively constant while the content of ZO-2 at the nucleus suffered drastic variations with time [47].

In contrast to sparse cultures, in confluent monolayers, blocking the nuclear exportation of ZO-2 with a PKCε permeable inhibitor peptide did not trigger the nuclear accumulation of ZO-2, indicating that in confluent cultures, ZO-2 travels directly to the plasma membrane without stopping at the nucleus [44].

In epithelial monolayers cultured in media with a low concentration of Ca^2+^, ZO-2 appeared to be diffusely distributed in the cytoplasm and accumulated at the nucleus [39] (Figure 2A). If these cultures were transferred to normal Ca^2+^-containing medium, TJs assembled and TER developed [49]. However, if the calcium-switch was done in ZO-2 silenced monolayers, a significant delay occurred in the arrival to the cell borders of ZO-1, occludin, and E-cadherin [17]. Integration of ZO-2 to the TJ is triggered by a signaling cascade that starts at the plasma membrane with the activation of the Ca^2+^-sensing receptor (CaSR) [41], a G-protein coupled receptor [50]. CaSR signals through the Gα_q/11_ subunit, which activates phospholipase C (PLC). PLC hydrolyzes PIP2 to diacyl glycerol (DAG) and inositol triphosphate (IP3). The latter induces the release of Ca^2+^ from the endoplasmic reticulum, while DAG leads to the activation of nPKCε. This kinase phosphorylates the kinase WNK4 that binds and phosphorylates ZO-2 in serine residues, triggering its disassembly from 14-3-3 and its relocation to TJs [41].

## 5. Interaction of ZO-2 with Nuclear Proteins

**Figure 3:**
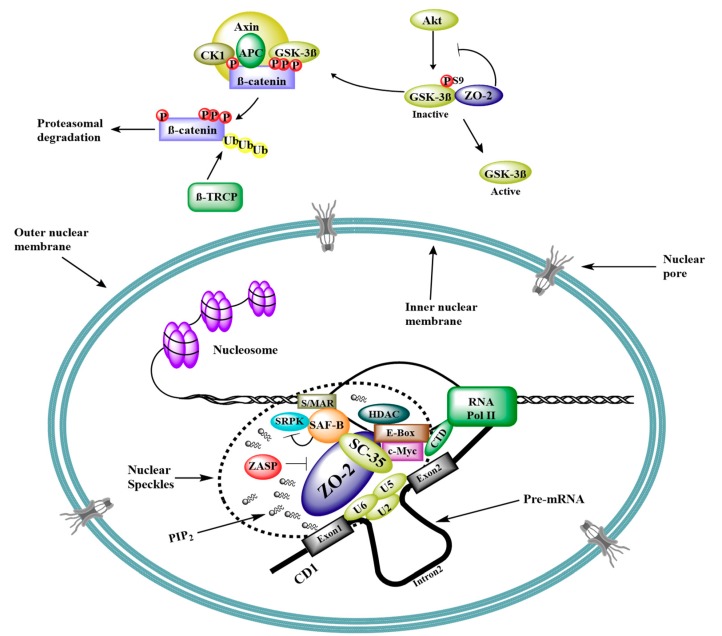
Schematic diagram of ZO-2 as a scaffold for transcription and splicing. ZO-2 expression blocks GSK-3β inhibitory phosphorylation at Ser9. GSK-3β phosphorylates β-catenin and targets it for ubiquitination and proteasomal degradation. Consequently, β-catenin entry into the nucleus and transcriptional activity is inhibited. At the nucleus, ZO-2 localizes in speckles associated to PIP2 and ZASP and inhibits transcription of CD1 through its interaction with an E-box in the promoter region, mediated by c-Myc transcription factor. In addition, ZO-2 is proposed to participate in mRNA splicing due to its association with the essential splicing protein SC-35 and the scaffold attachment factor SAF-B. S/MAR, scaffold/matrix attachment región; P, phosphate; Ub, ubiquitin; U2, U5, and U6, splicing ribonucleoproteins; PIP2, phosphatidylinositol (4,5)-bisphosphate.

ZO-2 is present in the nuclear matrix interacting with lamin B1 and actin [51]. Nuclear ZO-2 concentrates at speckles, which are active places that coordinate transcription and splicing. Accordingly, nuclear ZO-2 co-localizes with the essential pre-mRNA splicing protein SC-35 [39], the speckle protein ZASP [52], and the C-terminal portion of scaffold attachment factor-B (SAF-B) [40]. The latter is involved in transcriptional repression (for review on SAFB1/SAFB2 see [53]), associates with E-boxes in DNA [54,55], recruits histone deacetylases allowing histones to wrap the DNA more tightly [55,56], and binds and inhibits SRPK1 [57] (Figure 3).

In addition, a proteomic analysis revealed the presence of ZO-2 in a complex derived from an immunoprecipitation (IP) of SRm160 from a HeLa cell nuclear extract. SRm160 is a serine/arginine repeat nuclear matrix protein that promotes splicing and stimulates 3′-end processing [58].

In HEK293 cells, liquid chromatography-tandem mass spectometry (LC-MS/MS) revealed the presence of ZO-2 in SIRT7 immunoisolates [59]. SIRT7 is a nucleoli enzyme that associates with RNA Pol I machinery and is required for rDNA transcription. This is noteworthy, since ZO-2 does not co-localize at the nucleoli with nucleolin [39]. LC-MS/MS has also shown the presence of ZO-2 in a soluble protein complex with: (a) Ubinuclein [60], a nuclear protein that enhances the stability and function of human histone H3.3 chaperone complex HUCA (HIRA/UBN1/CABIN1/ASF1a) [61] and that in confluent epithelial cells interacts with cingulin and ZO-1 at TJs [62]; (b) WDR36/UTP21 [60], a protein required for the biogenesis of 18S ribosomal RNA [63]; (c) ZMYM3 [60], a chromatin-interacting protein that promotes DNA repair by homologous recombination [64]; (d) the estrogen-related receptor α (ERRα) [60], a nuclear receptor that interacts with estrogen, activates reporters containing steroidogenesis factor 1 response elements [65], and modulates the activity of estrogen receptor α (ERα) in breast, uterus, and bone (for review see [65]); (e) MCM3, a protein involved in the initiation of eukaryotic genome replication [66]; and f) PUS7 [66], a pseudouridine synthase that enhances RNA stability [67].

Altogether, these observations suggest that nuclear ZO-2 forms part of a complex that couples chromatin structure to transcription and RNA processing.

## 6. ZO-2 as Repressor of Gene Transcription

Pull-down and gel shift assays revealed the interaction of ZO-2 with Fos, Jun, and C/EBP transcription factors. In addition, IP and immunolocalization experiments showed that ZO-2 associates with these transcription factors, both at the nucleus and the TJ region [68]. The role of ZO-2 in gene transcription was first studied in reporter gene assays with promoters regulated by AP-1 sites, revealing that ZO-2 downregulates these promoters in a dose-dependent manner [68]. Then, in reporter gene assays, ZO-2 was found to downregulate cyclin D1 (CD1) transcription in a dose-dependent manner, acting upon a region in the promoter that harbored an E-box. IP and chromatin IP (ChIP) assays further revealed that the transcription factor Myc and the histone deacetylase (HDAC) 1 bound to this E-box and formed a complex with ZO-2 to repress CD1 transcription. The over-expression of Myc increased ZO-2-mediated inhibition of CD1 transcription [55], whereas the speckle protein ZASP blocked this transcriptional inhibition [52] (Figure 3).

ZO-2 also represses gene transcription regulated by the Wnt signaling pathway, which leads to cell proliferation and un-differentiation. Reverse transcription quantitative PCR (RT-qPCR) showed that the endogenous expression of axin-2, a Wnt target gene, was repressed upon ZO-2 overexpression [42]. Moreover, with IPs of transfected and endogenous proteins, as well as proximity ligation assays, ZO-2 was found to bind to glycogen synthase kinase 3β (GSK-3β) [48], while ZO-2 overexpression decreased the amount of inactive GSK-3β phosphorylated at Ser9 [42] (Figure 3). This enzyme, which inhibits epithelial to mesenchymal transformation (EMT), phosphorylates slug transcription factor, inducing its subsequent degradation. Accordingly, an increase in GSK-3β phosphorylated at Ser9 was found in tumor cells associated with slug expression. This factor, which represses E-cadherin transcription, belongs to the snail family, and LC-MS/MS revealed that ZO-2 was present in slug IPs [69]. Likewise, affinity purification of epitope tagged proteins followed by MS (AP-MS) identified ZO-2 as an interacting partner of smug, another transcription factor of the snail family [70].

GSK-3β also phosphorylates β-catenin at Ser33, Ser37, and Thr41, leading to its ubiquitination and subsequent proteasomal degradation (Figure 3). Instead, when β-catenin is not degraded, it accumulates in the cytoplasm and is translocated into the nucleus where it forms a complex with T cell factor (TCF)/lymphoid enhancer family (LEF) transcription factor, which induces the transcription of a variety of target genes involved in cell cycle progression and EMT (for reviews see [71,72]). The effect of ZO-2 on the transcription of Wnt target genes was analyzed in two different Wnt reporter constructs, where luciferase expression was regulated either by artificial TCF/LEF binding sites or by the promoter of Siamois, a Wnt target gene. In both cases, dose-dependent repression was exerted by ZO-2. Moreover, the transcriptional activity regulated by artificial TCF/LEF binding sites increased in ZO-2 KD cells in comparison to parental cells, indicating that the absence of ZO-2 facilitated TCF-regulated transcriptional activity [73]. In agreement, in colon carcinoma cells, vitamin D3 promotes ZO-2 expression and inhibits the expression of a variety of β-catenin-TCF4 responsive genes [74].

The capacity of ZO-2 to repress gene transcription regulated by the Wnt pathway was studied in mice *in vivo*, where ZO-2 overexpression in the glomerulus was achieved by hydrodynamic transfection. Treatment with adriamycin, an anthracycline antibiotic, produced a nephrotic syndrome triggered by activation of Wnt signaling, whereas ZO-2 overexpression in these animals controlled proteinuria and podocyte effacement and favored urea and creatinine clearance through a process that inhibited the expression of snail and increased the amount of nephrin and phosphorylated β-catenin in the glomeruli. This indicates that ZO-2 protects against podocyte injury induced by aberrant Wnt signaling activation [75].

The ability of ZO-2 to inhibit gene transcription regulated by the Wnt pathway is controlled by the intracellular localization of ZO-2. Thus, this repressive activity was not observed when a constitutively SUMOylated ZO-2 construct that could not enter the nucleus was employed. Instead, using a ZO-2 SUMOylation mutant (K730R) that exhibited a strong delayed nuclear export increased ZO-2 repressive activity on promoters regulated by TCF/LEF binding. Accordingly, a ZO-2 construct with forced nuclear localization due to fused NLS completely inhibited transcriptional activity mediated by TCF/LEF, even in dense cultures [48].

To address if ZO-2 repression of this transcriptional activity requires the presence of β-catenin, a LEF chimera lacking the β-catenin binding site and instead containing the Herpes virus VP16 transactivation domain (ΔNLEF-1-VP16) was employed. ZO-2 exhibited transcriptional repression with this LEF chimera in reporter assays done either with a synthetic promoter or with the Siamois promoter, thus demonstrating that ZO-2 inhibits TCF/LEF mediated gene transcription in a β-catenin-independent manner [48]. Nevertheless, ZO-2 does form a complex with β-catenin. This was demonstrated in co-IP and proximity ligation assays employing transfected and endogenous proteins. Moreover, pull-down assays with recombinant ZO-2 and β-catenin proteins revealed that ZO-2/β-catenin interaction was direct. These observations hence suggest that ZO-2 repressed TCF/LEF mediated transcriptional activity in both a β-catenin-independent and -dependent manner [48].

Yes-associated protein (YAP), the main downstream effector of the mammalian Hippo pathway that regulates cell size, proliferation, and differentiation (for review see [76]), docks the ubiquitin ligase β-TrCP to the β-catenin destruction complex in cells where the Wnt signaling pathway is inactive, while Wnt ligands dislodge YAP from this complex and trigger the nuclear accumulation of YAP [77]. YAP Ser127 phosphorylation by the large tumor suppressor (LATS) kinase of the Hippo pathway creates a 14-3-3 binding site, which allows the cytoplasmic retention of YAP. However, YAP phosphorylation at Ser381 by LATS primes YAP for a subsequent CK1 phosphorylation that triggers YAP ubiquitination and proteasomal degradation [78]. Instead, the inactivation of the Hippo pathway allows the entry of the un-phosphorylated YAP into the nucleus, where YAP acts as a cofactor for transcriptional enhanced associate domain (TEAD) transcription factors that promote EMT (for review see [76]). MDCK ZO-2 KD cells exhibit a nuclear accumulation of YAP even when grown at high density and display an increased transcriptional activity of a reporter driven by artificial TEAD-binding sites or by the promoter of connective tissue growth factor (CTGF), a YAP target gene, while transfection of ZO-2 reduces promoter activity in both parental and ZO-2 KD cells. Moreover, the absence of ZO-2 increased the amount of CTGF mRNA determined by RT-qPCR, while the overexpression of ZO-2 reduced CTGF mRNA in parental cells. Altogether, these results indicate that ZO-2 repressed the transcriptional activity mediated by TEAD [73].

The involvement of ZO-2 in gene transcription was also studied in human vascular smooth muscle cells (VSMC). ZO-2 was barely observed in isolated control coronary arteries, but was highly expressed after dilation with a balloon catheter in the tunica media, which is composed of SMC, and in the intima, which is made up of endothelial cells. In contrast, the transcription factor stat1 was inversely regulated upon dilation injury. In primary cultures of human VSMC, ZO-2 silencing increased the nuclear and cytosolic expression of stat1 and its mRNA level. When these ZO-2-silenced VSMC cells were transfected with a luciferase reporter plasmid under the control of an interferon stimulated response element (ISRE), to which stat binds, an induction of transcription was observed in comparison to control VSMC. These results suggest that ZO-2 in VSMC repressed stat1 transcriptional activity. The latter in VSMC inhibited the proliferation rate, which was important because an increased proliferation of medial VSMC is one of the hallmarks of negative vascular remodeling [79].

## 7. ZO-2 and Cell Proliferation

ZO-2 over-expression inhibits cell proliferation blocking, the progression of cells through the G1/S boundary of the cell cycle. This effect is due to the amino terminal segment of ZO-2, which contains the three PDZ domains of ZO-2 [42]. ZO-2 has also been found to block cell proliferation induced by YAP [80].

In MDCK cells, ZO-2 overexpression reduces the level of CD1 mRNA [55], but this effect is not observed when the cultures are synchronized. In these cultures, the level of CD1 mRNA is higher upon arrest after incubation with 0.1% serum than in proliferating cells cultured with 10% serum [42]. ZO-2 also inhibits cell proliferation by augmenting CD1 degradation in the proteasome induced by GSK-3β phosphorylation. Thus, at the S phase, GSK-3β phosphorylates CD1 at Thr286, inducing its exit from the nucleus, ubiquitination, and subsequent degradation in the proteasome [81]. ZO-2 overexpression diminishes GSK-3β inhibitory phosphorylation at Ser-9 and triggers CD1 degradation [42]. Accordingly, LiCl, which inhibits GSK-3β [82,83], blocks the decrease in CD1 protein content induced by ZO-2 overexpression [42].

ZO-2 KD in preimplantation mouse embryos [13] and in monolayers of MDCK cells [73] has no effect on the number of blastocyst cells or proliferating cells, respectively. Instead, ZO-2 silencing in the spontaneously immortalized kidney cell line mCCD_d1_, which displays highly differentiated properties of collecting duct principal cells, decreases cyclin D1 content and blocks cell cycle progression at G0/G1 [84]. This is noteworthy, because cells of the collecting duct show intense proliferation during embryogenesis but very low turnover in the adult kidney, suggesting that ZO-2 might play a regulatory role in this process.

In VSMC, ZO-2 silencing also inhibits cell proliferation, albeit through increased expression of stat1 transcription factor and activation of stat1 specific genes [79].

ZO-2 fused to a strong NLS of SV40, concentrates at the nucleus, increases cell proliferation, and triggers the expression of the M2-type isoenzyme of pyruvate kinase (PKM2) [85]. This enzyme, whose plasma concentration in cancer patients correlates with staging (for review see [86]), is involved in cancerous cell metabolism in a process known as the Warburg effect or aerobic glycolysis, where cancer cells take up high amounts of glucose and produce lactate even in the presence of oxygen. PKM2 catalyzes the last step of glycolysis, the conversion of phosphoenolpyruvate to pyruvate, with the concomitant production of ATP. Generation of ATP by PKM2, in contrast to mitochondrial respiration, is independent of oxygen and hence allows tumor cells to grow in hypoxic conditions. These results therefore suggest that the concentration of ZO-2 in the nuclei through the overexpression of PKM2 might confer a metabolic benefit to cancer cells.

## 8. ZO-2 in Apoptosis and Cell Degeneration

Apoptosis is a form of programmed cell death present in multicellular organisms. In contrast to necrosis, which is a traumatic cell death after acute cellular injury, apoptosis is a highly regulated and controlled process. Some factors, like Fas receptors and caspases, which are a family of cysteinyl-aspartate proteases that signal through a cascade, promote apoptosis, while proteins of the BCL-2 family inhibit apoptosis.

In human breast epithelial cells, upon apoptosis induction by staurosporine or anti-CD95/Fas antibody treatment, TJs are disrupted and ZO-2 is cleaved by caspases [87]. Caspase-mediated cell death requires cleavage of key proteins involved in cell survival, including PARP-1. This is an enzyme activated by DNA breaks that polyADP-ribosylates proteins. In response to mild DNA damage, PARylation favors DNA repair and cell survival, whereas severe DNA damage leads to apoptosis or necrosis by amplified PARP-1 activity, resulting in high donor nicotinamide adenine dinucleotide (NAD^+^) consumption and depletion of ATP pools (for review see [88]). In TK6 lymphoblastoid cells, after induction of apoptosis with hydroquinone, a metabolite of benzene involved in benzene-induced leukemia, ZO-2 is polyADP-ribosylated via interaction with PARP-1 in the nucleus and its expression is upregulated [89].

ZO-2 overexpression does not augment the percentage of early or late apoptotic epithelial cells in culture [42]. Instead, a tandem inverted genomic duplication of the wild type *TJP2* gene in humans leads to progressive non-syndromic hearing loss, accompanied by a change in the expression of BCL2 family genes that favors apoptosis. Although this change in gene expression was studied in the lymphoblast cells of affected individuals, it suggests that ZO-2 overexpression leads to increased susceptibility to apoptosis in inner ear cells and that this mechanism triggers adult-onset hearing loss in this kindred [4].

Ubiquitination regulates protein stability. In particular, lysine 48-linked polyubiquitination chains target proteins for degradation in the proteasome. Protein ubiquitination is counter-regulated by deubiquitinating enzymes, among which the ubiquitin specific proteases (USP) constitute the largest family. Although USP53 has been suggested to be catalytically inactive due to the absence of a critical hystidine residue, mambo mice carrying a point mutation in the catalytic domain of USP53 exhibit rapid progressive hearing loss. USP53 is broadly expressed in the inner ear, where it interacts with ZO-1 and ZO-2. In mambo mice, biotin tracer assays indicated that TJs of the stria vascularis were not affected, while hair cell degeneration preceded the endocochlear potential decline. Moreover, the outer hair cells of these mice evaded degeneration in organ culture, indicating that the unfavorable extracellular conditions that these cells encountered *in vivo* in the mambo mice promoted their degeneration. These observations hence suggest that the inactivation of USP53 promoted the degradation of ZO-1/ZO-2, triggering a disturbance of cochlear homeostasis that resulted in hair cell degeneration and apoptosis [90]. Moreover, in humans, cholestasis and hearing loss was found to correlate with a truncating variant in USP53 [91].

YAP, through its PDZ binding motif, interacts with the first PDZ domain of ZO-2; this interaction facilitates the nuclear localization of YAP. Nuclear YAP exerts a pro-apoptotic function determined by the appearance of a 89 kDa PARP-1 fragment characteristic of caspase-3 and -7 cleavage. The amount of this PARP-1 fragment decreases when ZO-2 expression is silenced, suggesting that ZO-2 facilitates the apoptotic function of nuclear YAP [80,92].

## 9. ZO-2 Associates with the Cortical Actomyosin Ring and Regulates Cytoarchitecture

In epithelial cells, F-actin associated with the apical junctional complex appears as a thin band that surrounds the apical margins of the cells, also known as the cortical ring of actin. Myosin II staining follows that of actin, but is more punctate and more concentrated at the tricellular junctions [93].

ZO-2 directly interacts with actin, but is not a F-actin cross-linking protein [10]. ZO-2 also binds to 4.1 [94], a cytoskeleton protein that stabilizes spectrin–actin interactions and associates with ankyrin (for review see [95]).

ZO-2 at the cell border is associated with the integral TJ protein JAM-A and the submembranous protein afadin. PDZ-GEF1 binds to this complex and stimulates the activity of the GTPase Rap2c, which inhibits RhoA. Consequently, in JAM-A-deficient cells, the activity of RhoA and the phosphorylation of myosin light chains are enhanced [96]. Moreover, the lack of this complex, which inhibits RhoA signaling, explains the engrossment of the perijunctional ring of actomyosin and punctate distribution of myosin II, which are observed in MDCK cells without ZO-1 and ZO-2 [93].

However, it is noteworthy that, in ZO-1KO/ZO-2KD mammary Eph4 cells, AJs are organized in a fragmented manner containing E-cadherin and actin but lacking myosin-II. In these cells, transfection of either ZO-1 or ZO-2 restores myosin integration and the establishment of regular AJs through a process mediated by Rho activation [97]. The differences in myosin II organization observed in MDCK and Eph4 cells which do not express ZO-1/ZO-2 could be due to the cellular context, since, in contrast to MDCK cells [9], Eph4 cells do not express ZO-3 [98].

In MDCK cells lacking ZO-1 and ZO-2, the change in the appearance of actin and myosin is accompanied by expansion of the apical domain, cell border recruitment of phosphomyosin light chain and Rho kinase 1 (ROCK-I), and contraction of the actomyosin ring [93]. In accordance, atomic force microscopy revealed that the lack of ZO-1 and ZO-2 increased the tension of the apical domain due to elevated myosin II ATPase activity [99]. In addition, in MDCK cells with shRNAs targeting both ZO-1 and ZO-2, the normal convoluted pattern of cell–cell contact becomes much more linear, making the overall cell shape more polygonal [93]. This change does not happen in ZO-2 KD [100] and KO [101] cells, but is clearly observed in ZO-1 KD [100] and KO [101] cells, and can also be attained in control cells after treatment with blebbistatin, an inhibitor of myosin II [101]. Moreover, excessive expression of ZO-1 in ZO-1 KO cells induces an intensive contoured shape of cell–cell junctions. Taken together, these observations indicate that the change from tortuous to linear contacts present in the double ZO-1 and ZO-2 KD is due to the lack of ZO-1 and depends on an actomyosin-generated force.

Structured illumination microscopy of ZO-1 reveals two conformations of the protein, stretched and folded. The latter relies on the interaction between the extreme C-terminal segment and the PDZ3, SH3, U5, and GuK domains of ZO-1. The conformational state of ZO-1 depends on its hetero-dimerization with ZO-2 and the tension of the actomyosin cytoskeleton. Thus, upon ZO-2 depletion, ZO-1 is maintained in the stretched conformation only if the actomyosin tension is high, like when cells are grown on glass coverslips. However, ZO-1 acquires a folded conformation if ZO-2 is absent and actomyosin tension is reduced by treatment with blebbistatin, or in conditions of reduced substrate stiffness (e.g., 3D cultures in Matrigel). In its folded conformation, ZO-1 cannot associate with occludin or recruit the transcription factor zonulin to the membrane, hence, this condition promotes the expression of zonulin target genes like cyclin D1 and proliferating cell nuclear antigen (PCNA) [102]. These observations suggest that, in a condition of low tension due to low substrate stiffness, like the one encountered by epithelial tissues *in vivo* (for review see [103]), the presence of ZO-2 is needed to maintain ZO-1 in a stretched conformation capable of binding to integral TJ proteins and to transcription factors like zonulin, which regulates the expression of genes involved in cell proliferation and differentiation.

**Figure 4:**
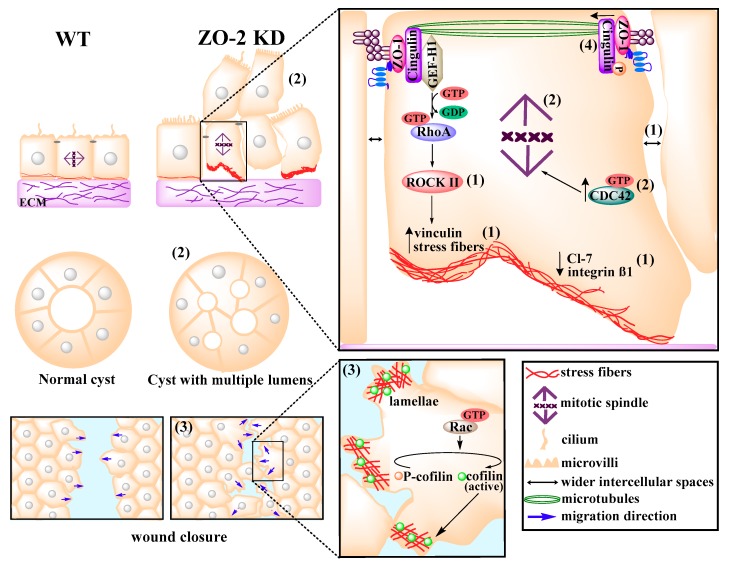
ZO-2 modulates cell size and tissue architecture. Absence of ZO-2 induces cell hypertrophy and development of an atypical cytoarchitecture characterized by: (**1**) Wider intercellular spaces and the detachment of cells from the substrate accompanied by a reduced expression of claudin-7 and integrin β1, an increased recruitment of vinculin to focal adhesions, and the proliferation of stress fibers induced by RhoA/ROCK-II activation; (**2**) an augmented activation of CDC42 that triggers the disorientation of the mitotic spindle and leads to the growth of cells on top of each other in 2D and 3D cultures and then to the formation of multiple lumens per cyst; (**3**) the activation of Rac and cofilin, which promote the formation of multiple lamellipodia, reduce directional persistence, and increase wound closure; and (**4**) the disappearance of a planar network of microtubules at the cell border.

ZO-2 alters the cytoarchitecture of epithelial monolayers, as the absence or reduction of ZO-2 content widens the intercellular spaces and detaches regions of the monolayer from the substrate [17,18]. In addition, decreased expression of integrin β1 and claudin-7, which localizes along the basolateral membrane in association to integrin β1, is observed in ZO-2 KD cells. Lack of ZO-2 induces recruitment of vinculin to focal adhesions and proliferation of stress fibers associated with increased activation of RhoA and ROCK-II [18] (Figure 4). Likewise, treatment of scirrhous gastric cancer cells with transforming growth factor β (TGFβ), whose signaling is associated with the invasion of cancer cells, decreases ZO-2 expression and upregulates the activity of RhoA and myosin phosphorylation [104].

ZO-2 KD cells also exhibit a profusion of lamellae mediated by an increase activity of Rac and cofilin. Rac promotes random cell migration in opposition to directional migration; accordingly, although ZO-2 KD cells move more than parental cells and close wounds more efficiently, they display reduced directional persistence [18] (Figure 4).

ZO-2 KD MDCK cells lose the microtubules that form a non-centrosomal network organized as a planar apical structure and display diminished phosphorylation of cingulin as well as a reduced interaction of cingulin with β-tubulin. ZO-2 favors the phosphorylation of cingulin by the adenosine monophosphate-activated protein kinase (AMPK), which allows microtubules/cingulin interactions at the apical junctional region. In addition, in ZO-2 KD monolayers, some cells grow on top of each other, while cysts of ZO-2 KD cells formed in the extracellular matrix Matrigel display multiple lumens instead of a single lumen per cyst. These aberrations might be due to the disorientation of the mitotic spindle, since in ZO-2 KD cells, the percentage of cells with mitotic spindles parallel to the substrate surface (0°–10°) decreases [18]. ZO-2 KD monolayers also display a reduced number of cilia [73] and more CDC42 activation [18], which controls spindle orientation [105] and centrosome polarization toward the direction of cell migration [106] (Figure 4). Taken together, these observations suggest that ZO-2 affects centrosome function.

The centrosome is a microtubule-organizing center composed of a centriole pair surrounded by pericentriolar material. In addition, centrioles template the formation of primary cilia in non-cycling cells. The mother centriole has sub-distal appendages needed for the organization of the cellular microtubular network and distal appendages involved in docking the centriole to the plasma membrane during ciliogenesis. Recently, using a proximity-dependent biotinylation assay to detect proteins interacting with centrosome and cilium proteins, ZO-2 was found to be associated with [107]: The sub-distal appendage proteins of the mother centriole CEP128, which regulates signaling at the primary cilium [108], and its associated protein cenexin [109] required for centrosome positioning in interphase cells, microtubule stability for proper spindle orientation during mitosis, and the formation of single lumens in cysts [110]; CEP135, a centriole assembly protein that regulates centriole duplication [111]; and centriolin, a protein of maternal centrioles required for the final stages of cytokinesis and entry into S phase [112].

## 10. ZO-2 as a Modulator of Cell Size

ZO-2 silencing in MDCK cells produces an increase in cell size also known as hypertrophy, where 67% of cells have a cell diameter of 55–60 nm instead of the 35–40 nm present in 73% of parental cells [73]. Hypertrophy has been associated with the activation of cyclin D without the concurrent upregulation of cyclin E, which triggers an increase in the physical growth of cells in early G1 and delayed entry into the S phase [113,114]. In ZO-2 KD cells this appears to be the case, as cyclin D1 content is increased, while entry into the S phase exhibits a delay [73].

In ZO-2 KD cells, hypertrophy is also due to inhibition of the Hippo pathway and activation of mTOR signaling [73]. The absence of ZO-2 induces the concentration of YAP at the nucleus and its transcriptional activity that promotes the expression of the catalytic pik3cb subunit of phosphatidylinositol 3-kinase (PI3K) and, through an miRNA-dependent process, blocks the translation of the phosphate and tensin homologue (PTEN). Consequently, the concentration of phosphatidylinositol(3,4,5)-trisphosphate (PIP3) increases and recruits PDK1 and AKT to the plasma membrane, triggering the phosphorylation and activation of AKT and the subsequent phosphorylation of mTORC1 and its downstream target, the ribosomal protein S6 kinase 1 (S6K1), which increases protein synthesis. Altogether, this leads to an increased protein/DNA ratio that produces hypertrophy [73], in agreement with previous observations in renal cells where hypertrophy appears to be a result of an increase in the protein synthesis/degradation rate [115,116]. Moreover, in renal compensatory hypertrophy induced by the removal of one kidney or uninephrectomy, the size of cells in the proximal tubules of the remaining kidney increased, while the expression of ZO-2 decreased and YAP accumulated in the nucleus. These results hence show that both *in vivo* and *in vitro* ZO-2 silencing correlates with cell hypertrophy.

## 11. ZO-2 as a Tumor Regulator Protein

Several lines of evidence suggest that ZO-2 is a tumor suppressor protein. First, ZO-2 is highly similar to Disc-Large (DLG) (amino segment with PDZ domains = 63% similarity, SH3 domain = 59% similarity, and GuK domain = 50% similarity), which acts as a tumor suppressor in the larva imaginal discs of Drosophila [6]. Second, some viral oncogenic proteins target ZO-2. Thus, the E4 region-encoded ORF1 (E4-ORF1), the primary oncogenic determinant of adenovirus type 9 that elicits mammary tumors in animals, associates through its PDZ binding motif with the first PDZ domain of ZO-2 and this interaction results in the aberrant sequestration of ZO-2 within the cytoplasm [117]. Likewise, E6 proteins from cancer-causing human papillomavirus (HPV)-16, -18, -31, -51, and -70 have a PDZ binding motif on their extreme carboxy termini that interacts with ZO-2. Instead, E6 proteins from HPV-66 or -40, which are, respectively, rarely or never associated with cancer, do not bind to ZO-2 [118]. E6/ZO-2 interaction appears to stabilize ZO-2, as ablation of E6 expression in HeLa cells reduces ZO-2 levels [118], whereas the expression of E6 from HPV-16 in the skin of transgenic female mice (K14E6) upregulated the expression of ZO-2 in the epidermis; and in monolayers of MDCK cells, transfection of E6 from HPV-16 blocked ZO-2 protein decay [119]. This is noteworthy because E6 proteins from high-risk HPV are known to target degradation of the cell–cell adhesion proteins Dlg [120], MAGI [120], MUPP1 [121], and Scribble [122]. E6 also triggers the mislocalization of ZO-2 as in MDCK cells transfected with E6, ZO-2 disappears from the cell borders and, in some cells, accumulates in the nucleus [119]. In this respect, in testicular carcinoma *in situ* and in bronchopulmonary cancers, ZO-2 disappears from the cell borders and stains the cytoplasm. This aberrant ZO-2 localization correlates with disruption of the blood–testis barrier [123] and the invasive properties of lung cancer cells *in vitro* [124].

c-Src, a non-receptor tyrosine kinase which is the cellular version of v-Src, the oncogene product of Rous sarcoma virus, is frequently overexpressed and active in various carcinomas. c-Src phosphorylates diverse cellular proteins involved in signaling pathways which lead to cancer progression. In MDCK cells, v-Src transfection opens the TJ, which is determined by a profound decrease in TER, and induces tyrosine phosphorylation of ZO-1 and ZO-2 [125]. Moreover, by proteomic analysis using the c-Src SH2 domain as the ligand, ZO-1 and ZO-2 were identified as binding proteins to both c-Src and C-terminal Src kinase (Csk). The latter phosphorylates and negatively regulates c-Src. Therefore ZO-1 and ZO-2 appear to function as scaffolding, regulating c-Src’s transformation ability [125].

The *TJP2* gene has six alternatively spliced transcripts encoding various isoforms. In normal epithelia, expression was studied in two isoforms of ZO-2: ZO-2A and ZO-2C, which are respectively transcribed from the downstream promoter P_A_ and the upstream promoter P_C_. The amino terminal region of ZO-2A has a 23-amino acid segment that is absent in ZO-2C [126,127]. In human pancreatic ductal adenocarcinoma and in breast ductal adenocarcinoma, ZO-2A isoform is absent, whereas in colon and acinar prostate adenocarcinomas, ZO-2 isoforms A and C are both expressed [128]. Loss of ZO-2A in pancreas ductal adenocarcinoma is not due to mutation, lack of transcription factors, or methylation of the immediate promoter, although methylation of the P_A_ promoter inactivates the promoter at the late stages of tumor development [126]. The sequence of promoter P_A_ reveals that ZO-2A transcription is regulated by numerous Sp1 sites [126].

Among *TJP2* transcripts, one with a longer exon which includes an AluYc sequence (*TJP2*-Alu transcript) was described. Alu proteins are retrotransposons that regulate gene expression patterns. *TJP2*-Alu transcript expression is not tissue specific, although its highest expression is in the testis. *TJP2*-Alu transcript is more highly expressed in normal tissues compared to tumors of the liver, uterus, and breast, however, colon tumor tissue displays a strong expression of *TJP2*-Alu transcript not present in normal tissue, making it a potential biomarker for colorectal cancer diagnosis [129].

The expression of ZO-2 is silenced in several carcinomas, including breast [128,130] and pancreas [127], as well as in a hypoxia-resistant cancer cell lines derived from a scirrhous gastric carcinoma [131]. In patients with incompletely enhancing glioblastoma multiforme (GBM), who have a longer survival rate, ZO-2 is expressed at higher levels than completely enhancing GBM, which is associated with shortened survival [132].

17β-estradiol (E2) and its receptors are risk factors for the initiation and progression of endocrine-related cancers, like those of the breast, prostate, ovarian, and endometrial cells (for reviews see [133,134]). In ovariectomized mice, E2 decreased the expression of ZO-2 in mouse epidermis and, although progesterone induced ZO-2 overexpression, it could not overturn ZO-2 silencing induced by E2 [119].

TGFβ1, which promotes the invasion of cancer cells, decreases the expression of ZO-2 in scirrhous gastric cancer cells [104]. Vascular endothelial growth factor-A (VEGF-A) in pancreas cancer cells increases cell motility and decreases ZO-2 expression [135]. Contrarily, vitamin D3, which promotes the differentiation of colon carcinoma cells, induces the expression of ZO-2 [74].

In summary, ZO-2 could be viewed as a tumor suppressor protein due to its structural similarity with known tumor suppressor proteins and considering that it is a target of oncogenic kinases, is silenced in various carcinomas, and exerts transcriptional repression on genes involved in cell proliferation and EMT. However, since oncogenic HPVs upregulate ZO-2 expression and forced nuclear accumulation of ZO-2 increases cell proliferation and confers metabolic benefit to cancer cells, we think ZO-2 might be considered as a tumor regulator rather than a tumor suppressor.

## Figures and Tables

**Table 1:** ZO-2 mutations and consequences.

Species	Disease	Alteration	Location within ZO-2	Features of Disease	Reference
Human	ADNSHL	A112T	PDZ1		[19]
		T1188A	TEL		
		G694E	GuK		[20]
		Genomic duplication at chromosome 9p13.3-q21.13			[4]
	Familial hypercholanemia	V48A	PDZ1	Patients with itching, elevated bile acid concentration and fat malabsorption. TJP2 mutation combined with bile acid Coenzyme A (BAAT) mutation	[21]
	Intrahepatic cholestasis	I875T	GuK	71-year-old patient	[22]
		R322W	PDZ2	Disease since early infancy	
		A256T	ZO-2 deficiency		[23]
		S296A			
		Y261S			
		A454G			
		A664S			
		Q318G			
		A632fs			
		S1136A			
		T880S			[24]
		N814Q		Disease since early infancy that leads to hepatocellular carcinoma	[25]
		A273fs			[26]
		Exon 18 deletion			[27]
		T62M	PDZ1	Intrahepatic cholestasis of pregnancy (ICP)	[28]
		T626S	SH3		
Mouse		ZO-2 KO		Reduced male fertility	[16]

ADNSHL: Autosomal dominant non-syndromic hearing loss.
